# Experiences with Gluten-Free Bread: A Qualitative Study Amongst People with Coeliac Disease Participating in a Randomised Controlled Trial

**DOI:** 10.3390/foods12234338

**Published:** 2023-12-01

**Authors:** Lisa Garnweidner-Holme, Monica Hellmann, Christine Henriksen, Elisabeth Austad, Solveig Ivara Watters, Line Gaundal, Knut E. A. Lundin, Mari C. W. Myhrstad, Vibeke H. Telle-Hansen

**Affiliations:** 1 Department of Nursing and Health Promotion, Faculty of Health Sciences, Oslo Metropolitan University, P.O. Box 4 St. Olavs Plass, 0130 Oslo, Norway; eaustad@hotmail.com (E.A.); solveigi@oslomet.no (S.I.W.); gaundall@gmail.com (L.G.); mmyhrsta@oslomet.no (M.C.W.M.); vtelle@oslomet.no (V.H.T.-H.); 2 Det Glutenfrie Verksted, Nordseterveien 26A, 1176 Oslo, Norway; monica@detglutenfrieverksted.no; 3 Department of Nutrition, Institute of Basic Medical Sciences, University of Oslo, Boks 1072 Blindern, 0316 Oslo, Norway; christine.henriksen@medisin.uio.no; 4 K. G. Jebsen Coeliac Disease Research Centre, University of Oslo, Boks 1072 Blindern, 0316 Oslo, Norway; knut.lundin@medisin.uio.no; 5 Institute for Clinical Medicine, University of Oslo, P.O. Box 1171 Blindern, 0318 Oslo, Norway; 6 Department of Gastroenterology, Oslo University Hospital, Rikshospitalet, 0424 Oslo, Norway

**Keywords:** coeliac disease, bread, randomised controlled trial, fibre-rich

## Abstract

Background: Whole-grain bread can be an important source of fibre for people with coeliac disease (CeD) who must adhere to a gluten-free diet and avoid consuming wheat, rye and barley. Gluten-free bread frequently has a lower nutritional quality and different texture relative to gluten-containing counterparts. Objective: The aim was to investigate experiences with gluten-free bread amongst people with CeD prior to and during a randomised controlled trial (RCT). Design: We conducted individual interviews with 10 people with CeD participating in a RCT that aimed to investigate the effects of fibre-rich gluten-free products on metabolic regulation in people with CeD compared with benchmark gluten-free products. Five participants were in the control group (benchmark gluten-free bread) and five participants in the intervention group (fibre-rich gluten-free bread). The fibre-rich gluten free bread was formulated and prepared by the project group. The benchmark gluten-free bread was commercially available. The RCT lasted for four weeks. Interviews were conducted digitally between October 2021 and January 2022 and were thematically analysed. Results: Participants in both groups appeared to avoid bread prior to the study, primarily due to the poor taste and chewy consistency of the available bread in food stores and bakeries. Participants preferred the fibre-rich intervention bread as opposed to the available bread in the food market. However, participants had to become accustomed to eating the fibre-rich whole-grain bread during the study, since they avoided eating store-bought bread that they experienced chewy and not filling. Conclusions: Participants asked for fibre-rich gluten-free bread products that are satiating and have a good texture. Palatable gluten-free bread products might be an important source of fibre for people with CeD.

## 1. Introduction

People with coeliac disease (CeD) may have challenges meeting the daily recommendations for fibre intake [1]. Fibre-rich gluten-free bread might contribute to a healthier diet for people with CeD. However, a growing proportion of the literature has indicated the lower nutritional quality of gluten-free products and a lower average satisfaction with such products related to taste and texture amongst people with CeD [2,3,4,5]. Because of their viscoelastic properties, gluten proteins contribute to a puffy and chewy texture in the dough. The lack of these properties may hinder the production of palatable gluten-free products. People with CeD frequently demand tastier gluten-free products [6,7,8]. We previously compared the macronutrient content of 423 unique gluten-free products with 337 equivalents with gluten on the Norwegian food market. The gluten-free products contained less protein and fibre and higher amounts of saturated fat, carbohydrates and salt compared to the gluten-containing products [4]. Gluten-free products normally contain starches and refined flours, providing a low fibre content [9]. Bread is an important source of dietary fibre in many food cultures. CeD is a chronic autoimmune disease in which villous atrophy in the small intestine is triggered by gluten exposure [10]. CeD is one of the most common lifelong food-related disorders [10] and the prevalence of CeD is increasing worldwide. In Norway, the prevalence of CeD is estimated to be between 1 and 2%, which means that between 50,000 and 100,000 people have the disease [11]. The only currently available treatment for CeD is strict adherence to a gluten-free diet [12]. Even small amounts of gluten (i.e., 50 mg) can be harmful to people with CeD [13]. Untreated CeD leads to gastro-intestinal symptoms, with or without systemic manifestations [14]. Since people with CeD have to follow a gluten-free diet their whole lives, it is important to find solutions for the challenges experienced by people with CeD who follow a gluten-free diet [4,6,7,15,16]. Difficulties adhering to a gluten-free diet may arise for several reasons. For instance, in a Canadian national survey regarding the impact of a gluten-free diet, 44% of 2681 people with CeD had difficulties following the diet [17]. Various studies have found that gluten-free products are more expensive than their gluten-containing counterparts [16,18,19]. People with CeD have also reported dissatisfaction with the availability of gluten-free products in food stores, bakeries and restaurants [7,8,20,21,22,23].

Participants in this qualitative study were part of the GRAIN study, a randomised controlled trial (RCT) that aims to investigate the effects of fibre-rich gluten-free products on metabolic regulation in people with CeD compared with benchmark gluten-free products. Ten participants from the GRAIN study participated in qualitative, individual interviews. Given the importance to develop palatable, fibre-rich products for people with CeD, the primary objective of the interviews was to investigate participants’ experiences with fibre-rich gluten-free bread. This article focuses on participants’ experiences with bread during the GRAIN study, as participants were most concerned about bread during the interviews.

## 2. Materials and Methods

### 2.1. Study Setting and Sampling

This qualitative study was conducted amongst 10 out of 30 participants in the GRAIN study [manuscript in preparation]. All of the participants in the GRAIN study had been diagnosed with CeD. Participants’ diagnoses of CeD were self-reported. The GRAIN study lasted for a total of four weeks, including a one-week run-in and a three-week intervention period. During the run-in, the participants consumed the following control products: six slices (approximately 35 g per slice) of a commercially available gluten-free bread (Brisk, Ålesund, Norway) (hereafter called ‘standard bread’) and one portion of corn flakes (60 g) (Det Glutenfrie Verksted, Oslo, Norway). Both products contained less than 6 g of fibre per 100 g. After the run-in period, participants were randomly assigned to either a control group or an experimental group. While the control group continued with the same products and amounts as those administered during the run-in period, the participants in the experimental group were given four slices of fibre-rich (>6 g fibre/100 g) bread (50 g), one roll (50 g) and one portion of muesli (60 g) (Det GlutenfrieVverksted, Oslo, Norway) daily. This bread was formulated and prepared by the project group. The study was performed remotely, meaning that all communications with the participants were conducted via online Zoom meetings or by e-mail and telephone. The food products were delivered to the participants’ homes. The GRAIN study was registered on Clinicaltrials.gov (NCT05135923). Ethical approval for the RCT and the qualitative interviews was obtained by the Regional Committees for Medical Research Ethics in South-East Norway (83004) and from the Norwegian Centre for Data Security (437313). The study was conducted in accordance with the Declaration of Helsinki, and the participants provided their written informed consent to participate.

All of the participants in the GRAIN study were recruited via a recruitment website, namely the Oslo Metropolitan University (OsloMet) website. The website was promoted via Facebook and Instagram. The Norwegian Association for CeD also promoted the website amongst its members. The inclusion criteria required participants to be men and women who had been diagnosed with CeD but who were otherwise healthy, aged between 18 and 65 years and of a normal weight (BMI 18.5–27 kg/m^2^). Participants’ weight was self-reported. All of the participants in the GRAIN study were female. The process of recruiting started in September 2021 and continued throughout November 2021. After the run-in period, the participants were randomly allocated to consume optimized gluten-free products (GFP) or benchmark GFP by a researcher not involved in the data collection. A more detailed description of the design, the nutritional contents of the study’s products, the recruitment process and the study’s participants will be presented in a manuscript in preparation.

### 2.2. Data Collection

The first five participants from the control group and the first five participants from the experimental group that finalized the GRAIN study were asked by the project group to participate in an individual interview. They were interviewed by the first author (a professor in health and nutrition communication) after the intervention (from October 2021 to January 2022). Five of the participants in this qualitative study were in the experimental group. The other five participants were in the control group receiving benchmark bread and corn flakes. The interviews adhered to a semi-structured interview guide developed by the multi-professional project group. The main themes in the interview guide were: (1) experiences with a gluten-free diet; (2) experiences with the products in the GRAIN-study; (3) experiences with participating in the GRAIN-study. The interview guide is attached as Supplementary Materials File S1. The first author pilot-tested the interview guide. The pilot test interview was included in the analysis, as only minor adjustments were made in the interview guide. The first author was not involved in the recruitment process and did not have any personal relationships with the participants prior to the study. The interviews lasted between 14 and 33 min and were conducted via Zoom. The recruitment process was conducted until we had achieved sufficient informational power, depending upon the quality of the interviews and the aim of the study [24]. We followed the consolidated criteria for reporting qualitative studies (COREQ) [25].

### 2.3. Analysis

The interviews were audiotaped and transcribed by the first author. The transcripts were compared with the audiotapes to ensure the accuracy of the transcription process. The analysis was performed by the first author and was guided by thematic analysis, according to Braun and Clarke [26,27]; this entailed the following steps: (1) becoming familiar with the data through the repeated reading of each informant’s transcripts; (2) generating initial codes (words or short phrases in the transcripts) that were relevant to the research questions; (3) organising codes into sub-themes; (4) arranging sub-themes into overarching themes; and (5) defining and naming the themes. A qualitative software program called NVivo (12.0) was used to identify codes and systematise sub-themes. Codes and sub-themes were labelled in two colours to distinguish between the control group and the experimental group. The first author discussed potential codes and themes with the last two authors.

## 3. Results

### 3.1. Background Information

Table 1 presents the relevant background information regarding the participants and their self-reported perceptions concerning managing a gluten-free diet. All of the participants were incidentally female. The mean age of the participants in the GRAIN study was 35 years. The time that had elapsed following a diagnosis with CeD varied from 2 to 22 years. When participants were asked about their general experiences with managing a gluten-free diet, they appeared to be capable of managing a gluten-free diet, especially some years after their diagnosis.

### 3.2. Participants’ Experiences with Bread Prior to and during the GRAIN Study

Barriers managing a gluten-free diet included insecurities in social settings and limited gluten-free products in food stores. Participants in both groups were motivated to participate in the GRAIN study to contribute to enhancing gluten-free products or to improve their digestive symptoms. Table 2 presents the sub-themes and main themes from the thematic analysis of participants’ experiences with bread prior to and during the GRAIN study.

### 3.3. Experiences with Bread Prior to the Study

The majority of the participants in both the control and experimental group indicated that they did not eat bread prior to the study. The main reason for avoiding bread was that they did not find bread in food stores or bakeries that they liked or could afford, as illustrated by the following statement by a participant in the control group who had been diagnosed with CeD four years ago:

‘I personally do not eat so much bread because I think that the products are so bad’.

(C6)

Participants stated that they did not like the taste and consistency of the available bread in food stores and bakeries and described products as ‘too chewy’ or ‘tasteless’. One participant had become accustomed to baking bread herself, as expressed in the following statement:

‘I have given up [laughing], with everything that is ready made. I just buy flour and make everything on my own. It is difficult to find something that saturates more than half an hour and that also tastes good. I have baked myself for the last 10 years’.

(C7)

Others indicated that it was difficult to bake gluten-free products.

### 3.4. Experiences with Standard Bread

Participants in both groups ate standard bread during the run-in; they mostly described the bread as tasteless and not satiating due to its consistency. One participant in the experimental group expressed the following: ‘The light bread tasted good, but I did not feel that it was that useful’. (E2)

When the interviewee asked what the participant meant by ‘useful’, the participant stated the following: ‘It looked like wheat bread, and it was not satiating’. (E2)

However, divergent experiences surfaced; for instance, one participant in the control group stated the following: ‘They were not my favourites. They were in the category “satiating, but didn’t taste good”’. (C7)

Some participants also enjoyed the taste of the standard bread.

### 3.5. Experiences with the Fibre-Rich Gluten-Free Bread

Participants in the experimental group preferred the fibre-rich gluten-free bread as opposed to the standard bread they were provided in the run-in period. They preferred the taste of the fibre-rich gluten-free bread and experienced enhanced satiety (related to perceived fullness) after eating this bread as opposed to the standard bread: ‘The bread both tasted good and was satiating.’ (E1)

However, some participants in the experimental group stated that they had to become accustomed to eating whole-grain bread during the study, as indicated by a participant who was diagnosed with CeD 22 years ago: ‘I had to get used to the whole grain bread, but I do not like bread that much. I think that the bread left some weird taste in the mouth. It was quite compact. I almost was saturated before I ate it.’ (E2)

Participants in the experimental group also preferred the fibre-rich gluten-free bread compared to bread from food stores and bakeries, as conveyed by the following participant who has adhered to a gluten-free diet for 17 years: ‘Bought bread is more rubbery and tough’. (E1) However, participants sometimes mentioned that the taste of the fibre-rich gluten-free bread was ‘strong’ and ‘unknown’.

### 3.6. Difficulties with Eating Bread during the Study

Participants in both the control group and the experimental group experienced difficulties eating four to six slices of bread each day, as they had not become accustomed to eating bread prior to the study: ‘I should eat 4 slices and a lot of cornflakes each day [laughing]. I didn’t eat bread usually and it did not feel healthy’. (E2)

Participants who experienced difficulties with eating the amount of bread required during the study period found it challenging to decide what type of bread spreads to use, since bread had not been part of their daily diet prior to the study. Some also experienced that the amount of bread was satiating to the degree that they did not manage to eat other foods, such as fish, meat, fruits and vegetables, to maintain a healthy diet.

### 3.7. General Motivations and Experiences Participating in the RCT

Participants’ involvement in the RCT was frequently motivated by a desire to contribute to the innovation of enhanced gluten-free bread in food stores. Others participated with the hope of improving their gastrointestinal symptoms. During the interviews, participants also discussed their general experiences of partaking in the RCT. Participants valued the digital performance of the study and outlined the advantages of having the study products delivered to their home. Experiences with taking clinical tests at home varied. Some participants found it difficult to take blood samples at home, whereas others did not. Participants acknowledged the effective communications and support from the study personnel during the study.

## 4. Discussion

Participants in the experimental group preferred the fibre-rich gluten-free bread due to taste, consistency and enhanced satiety. In line with other studies [6,7,22], participants in both the control and experimental groups asked for higher-quality gluten-free products. To our knowledge, this is the first study to explore experiences with gluten-free bread amongst people with CeD.

In many industrialised countries, bread constitutes a major component of one’s diet and the main source of one’s fibre intake [28,29]. Gluten proteins have unique viscoelastic and adhesive properties, giving the dough a puffy and chewy texture. Due to gluten’s importance in determining bread’s texture, it is challenging to produce gluten-free bread with the same taste and texture as gluten-containing bread. Aside from texture, our previous analysis of the nutritional quality of 66 gluten-free breads on the Norwegian market demonstrated that gluten-free bread had significantly higher amounts of total fat (59%), saturated fat (80%) and salt (11%) compared to their gluten-containing counterparts [4]. Similar results were uncovered in studies from Canada and the UK in which gluten-free staple foods contained less fibre compared to gluten-containing staple foods [2,30]. People with CeD frequently struggle to meet the daily recommendations for fibre [1]. Hence, fibre-rich gluten-free bread may contribute to increased fibre intake amongst people with CeD.

However, the production of fibre-rich gluten-free products is difficult. The fibre-rich gluten-free bread in this study was produced with pseudocereals. Pseudocereals (e.g., quinoa, amaranth, buckwheat) are nutritious and gluten-free ingredients [31]. However, the utilisation of pseudocereals is hampered by substances that yield a bitter taste, as outlined by our participants. As the growth of pseudocereal cultivation, particularly that of quinoa, remains largely restricted to the nations in which the pseudocereals originated [31], pseudocereals are expensive ingredients in many countries. For instance, due to the limited availability of pseudocereals in Norway, they are more expensive than gluten-containing cereals. Although participants in both the control and experimental groups in this study did not mention expensive products as a barrier in relation to managing a gluten-free diet, the price of gluten-free products was one of the most significant hindrances to maintaining a gluten-free diet in other studies [6,22,32]. Some participants in our previous study accepted the higher price because of the higher costs involved in producing gluten-free products for the food industry or because they received reimbursements from the government [7].

In the interpretation of our results, it must be acknowledged that most of the participants in the control and experimental group had adhered to a gluten-free diet for more than 10 years. They may have adapted to avoid bread and not tried new products on the market. The bread that participants in the experimental group in this study preferred contained pseudocereals that increase the nutritional quality of the bread; however, these ingredients could also contribute to a strong or unknown taste. Participants in both the control group and the experimental group had to become accustomed to eating bread during the study. The higher fibre content in the experimental bread could have led to increased perceived satiety compared to the standard bread. Furthermore, the participants had difficulties consuming varied bread spreads. Hence, informational material and recipes regarding how to prepare varied bread-based meals and sandwiches might increase the bread consumption of people with CeD.

### Study Limitations

This study was conducted with a small sample size. However, after interviewing 10 participants, we reached informational power to answer our research questions and similar sub-themes and themes were identified in the control and experimental group [33]. We also aimed to have equally as many interviews from each group. Involvement of an interdisciplinary project group, with both having their own experiences with living with a gluten-free diet, secured the trustworthiness of the results. The results from this study cannot be generalised, but might be transferable to participants with CeD who participate in similar RCTs. The study was conducted among adults. Not all age groups with CeD were represented. The interdisciplinary project group developed the interview guide and helped to interpret the data. Participants’ gluten-free diets were not examined. When interpreting the results, it must be acknowledged that participants’ involvement in the RCT was frequently motivated by a desire to contribute to the innovation of enhanced gluten-free bread in food stores. Hence, a selection bias towards people who did not like bread might have occurred. Participants were not asked about their preferences of gluten-free bread prior to the study, but their wish for more palatable gluten-free bread became clear during the interviews. Interviews were conducted via Zoom due to the COVID-19 pandemic. Although face-to-face communication and clinical tests might have been advantageous, participants in this and another study appreciated the digital performance of this study [34].

## 5. Conclusions

Participants in both the control and intervention group in this study requested fibre-rich gluten-free bread that was satiating and had a satisfying texture. They had to become accustomed to eating fibre-rich bread while participating in this study due to negative experiences with bread in stores. Palatable gluten-free bread products might be an important source of fibre for people with CeD. However, we need more knowledge about how pseudocereals can help to optimize gluten-free bread without adding a bitter taste. Participants appreciated the digital performance of the RCT study.

## Figures and Tables

**Table 1:** Background information regarding the participants and self-reported perceptions concerning managing a gluten-free diet prior to the study.

Participant	Years Since Diagnosed with CeD	Self-Reported Perceptions Concerning Managing a Gluten-Free Diet
1	17	‘I think that I succeed with a gluten-free diet.’
2	22	‘It is easier now.’
3	13	‘I think that it works out very good. I am very used to it. It is not something that I use to think about.’
4	2	‘I think that it works out very good.’
5	10	‘I think that it is ok, but I miss a lot. Mostly bakery and whole grain bread.’
6	4	‘It is very ok, because several people eat gluten-free at home.’
7	16	‘It is ok. I am very strict about it, because I was so sick in the start. It took a long time until I got healthy.’
8	15	‘It works out very well. I get so sick of it [gluten-containing food]. I only eat 100% gluten-free food and that’s not a problem.’
9	2	‘It varies. I succeed with the diet, but not in social settings.’
10	13	‘It works out very well. My parents handled the transition.’

**Table 2:** Sub-themes and main themes of participants’ experiences with bread prior to and during the GRAIN study.

Main Themes	Sub-Themes	Quote
Experiences with bread prior to the study	Does not like bread Asks for better bread in food stores and bakeries Bakes bread independently	“I personally do not like bread” (C6) “I wish to have better bread” (E2) “I have given up... with commercial bread… I bake my own bread” (C7)
Experiences with standard bread	Believes bread is not satiating due to consistency Does not like the taste	“The bread was too fluffy. It was not satiating” (E2) “I did not like the taste. It tasted nothing” (C5)
Experiences with fibre-rich gluten-free bread *	Prefers fibre-rich gluten-free bread due to taste Had to become accustomed to whole-grain bread	“I preferred the bread in the last three weeks. It tasted good” (E1) “I had to get used to eat whole grain bread during the study. It was very satiating” (E2)
Difficulties with eating bread during the study	Was not accustomed to eating bread prior to the study Experienced having to eat excessive bread	“I did not eat bread prior to the study because I don’t like the products in the stores” (E2) “It was difficult to eat so much bread because I usually don’t eat bread at all” (E2)
General motivation for and experiences of participating in the RCT	Motivated to participate for health-related reasons Motivated to participate to contribute to the research Motivated to participate to support product innovation Appreciated the opportunity to participate digitally Experienced challenges with blood samples at home Had positive communications with project members	“I participated to have less pain in my stomach” (C7) “I participated to contribute to research” (E2) “I wanted to support product innovation” (E4) “I sometimes struggled to take blood samples on my own” (C9) “We could always contact the project members to get help” (C1)

* This theme was only identified in the experimental group.

## Data Availability

The data presented in this study are available on request from the corresponding author. The data are not publicly available due to secure the privacy of the participants.

## Supplementary Materials

The following supporting information can be downloaded at: https://www.mdpi.com/article/10.3390/foods12234338/s1, File S1: Interview guide.
