# Supplementary material for: Experiences with Gluten-Free Bread: A Qualitative Study Amongst People with Coeliac Disease Participating in a Randomised Controlled Trial
Source: Foods. 2023 Dec 1;12(23):4338. doi: 10.3390/foods12234338 (PMC10706794; doi:10.3390/foods12234338)
Supplement: Supplementary file 1 [file foods-12-04338-s001.zip › foods-2701050-supplementary.pdf]

Interview guide

Interview_X

Duration of the interview:

General information about the interview (purpose, privacy)

Topic 1) Experiences with eating gluten-free

When were you diagnosed with celiac disease?

What are your experiences of managing a gluten-free diet?

Theme 2) Experiences with products in the Grain study

Can you tell us about your experiences with the products in the study?

What was it like following that menu in the study? Can you describe whether you had to make major changes to your diet?

How did you experience the products' taste and texture?

Can you tell me a little more about what it was like to eat and use the bread during the study?

Topic 3) Participation in the study

Why have you participated in the Grain study?

What was positive/negative about being a participant in the study?

What do you think about the duration of the study?

Did you feel that participation in the study has affected your health/symptoms related to celiac disease?
